# Supplementary material for: Drug repurposing for aging research using model organisms
Source: Aging Cell. 2017 Jun 16;16(5):1006–15. doi: 10.1111/acel.12626 (PMC5595691; doi:10.1111/acel.12626)
Supplement: Supplementary file 7 — Data S1 Zip‐Archive of all report cards. [file ACEL-16-1006-s007.zip › RC_CIR.pdf]

CIR

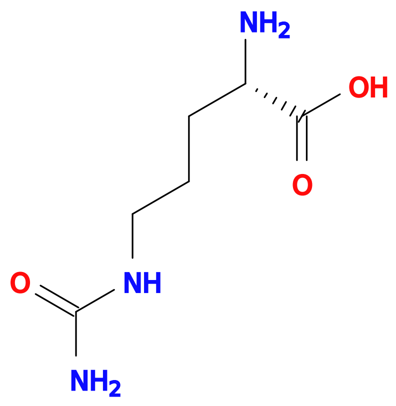

Database identifiers

ChEMBLCompound	CHEMBL444814
DrugBank	DB00155
CHEBI	16349
CHEBI	57743
ZINC	ZINC01532614
eMolecules	493823

Ranking

	Rank	Score
Drosophila	289/697	0.577
C. elegans	NA	NA

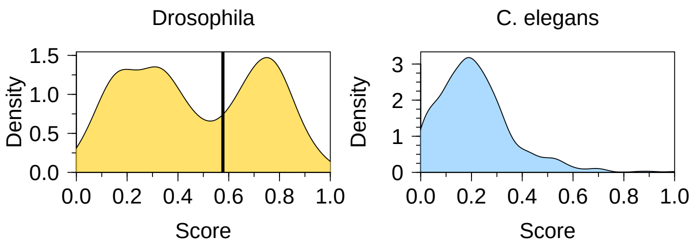

	Ageing implication	Domain conservation	Binding site conservation	Binding affinity	Bioavailability	Lipinski	Promiscuity	Purchasability	Drug approval	**Total**
Drosophila	0.792	0.918	0.946	0.65	(0.9)	0.0	-0.0	0.1	0.075	0.577
C. elegans	NA	NA	NA	NA	NA	NA	NA	NA	NA	NA

Names

- **L-citrulline zwitterion**
- (2S)-2-azaniumyl-5-(carbamoylamino)pentanoate

Roles

human metabolite, Escherichia coli metabolite, plant metabolite, Daphnia magna metabolite

Status

Approved drug (according to ChEMBL)	No
Classification (according to DrugBank)	approved, nutraceutical
Number of Rule of 5 violations	0
Binding affinity to original target in log units (RF-Score prediction)	5.62
Burns *C. elegans* bioavailability prediction	-6.39

Medical Information

Indicatation: Used for nutritional supplementation, also for treating dietary shortage or imbalance.
Mechanism of action: L-citrulline is converted to L-arginine by argininosuccinate synthase. L-arginine is in turn responsible for citrulline's therapeutic affects. Many of L-arginine's activities, including its possible anti-atherogenic actions, may be accounted for by its role as the precursor to nitric oxide or NO. NO is produced by all tissues of the body and plays very important roles in the cardiovascular system, immune system and nervous system. NO is formed from L-arginine via the enzyme nitric oxide synthase or synthetase (NOS), and the effects of NO are mainly mediated by 3',5' -cyclic guanylate or cyclic GMP. NO activates the enzyme guanylate cyclase, which catalyzes the synthesis of cyclic GMP from guanosine triphosphate or GTP. Cyclic GMP is converted to guanylic acid via the enzyme cyclic GMP phosphodiesterase. NOS is a heme-containing enzyme with some sequences similar to cytochrome P-450 reductase. Several isoforms of NOS exist, two of which are constitutive and one of which is inducible by immunological stimuli. The constitutive NOS found in the vascular endothelium is designated eNOS and that present in the brain, spinal cord and peripheral nervous system is designated nNOS. The form of NOS induced by immunological or inflammatory stimuli is known as iNOS. iNOS may be expressed constitutively in select tissues such as lung epithelium. All the nitric oxide synthases use NADPH (reduced nicotinamide adenine dinucleotide phosphate) and oxygen (O2) as cosubstrates, as well as the cofactors FAD (flavin adenine dinucleotide), FMN (flavin mononucleotide), tetrahydrobiopterin and heme. Interestingly, ascorbic acid appears to enhance NOS activity by increasing intracellular tetrahydrobiopterin. eNOS and nNOS synthesize NO in response to an increased concentration of calcium ions or in some cases in response to calcium-independent stimuli, such as shear stress. In vitro studies of NOS indicate that the Km of the enzyme for L-arginine is in the micromolar range. The concentration of L-arginine in endothelial cells, as well as in other cells, and in plasma is in the millimolar range. What this means is that, under physiological conditions, NOS is saturated with its L-arginine substrate. In other words, L-arginine would not be expected to be rate-limiting for the enzyme, and it would not appear that supraphysiological levels of L-arginine which could occur with oral supplementation of the amino acid would make any difference with regard to NO production. The reaction would appear to have reached its maximum level. However, in vivo studies have demonstrated that, under certain conditions, e.g. hypercholesterolemia, L-arginine could enhance endothelial-dependent vasodilation and NO production.
Retail: Medisca Inc.
L-citrulline powder - 1.01 USD / g

(Information from DrugBank)

Compound Target Characteristics

Argininosuccinate synthase

Best gene implication in ageing for this target family came from gene P09034 annotated in UniProt release 2014_02. Annotation GO 7568 (aging) was Inferred from Expression Pattern

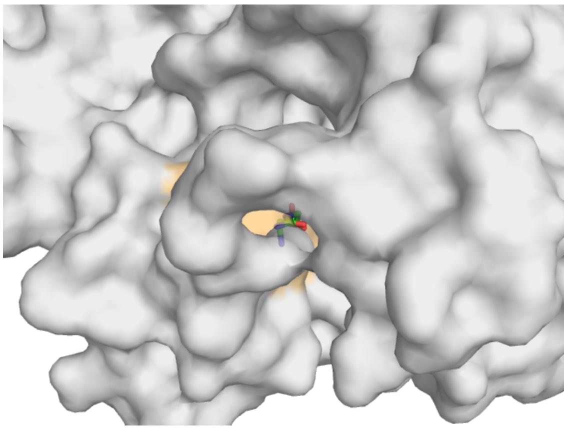

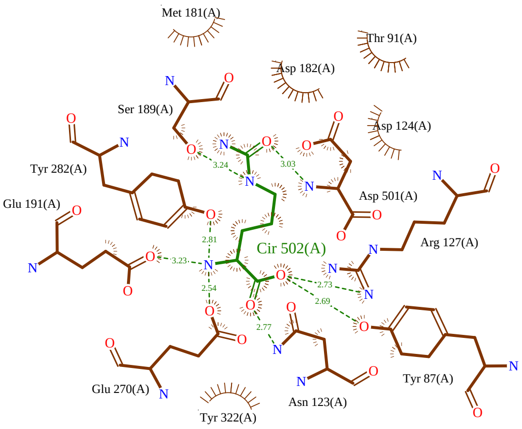

protein	amino acids contacts (binding site)													
PDB:2nz2:chainA:P00966	Y	T	S	N	D	R	S	M	D	S	E	E	Y	Y
tr:Q5T6L4:Q5T6L4_HUMAN	Y	T	S	N	D	R	S	M	D	S	E	E	Y	Y
sp:P00966:ASSY_HUMAN	Y	T	S	N	D	R	S	M	D	S	E	E	Y	Y
sp:P09034:ASSY_RAT	Y	T	S	N	D	R	S	M	D	S	E	E	Y	Y
sp:P16460:ASSY_MOUSE	Y	T	S	N	D	R	S	M	D	S	E	E	Y	Y
tr:Q3UJ34:Q3UJ34_MOUSE	Y	T	S	N	D	R	S	M	D	S	E	E	Y	Y
tr:J3QNG0:J3QNG0_MOUSE	Y	T	S	N	D	R	S	M	D	S	E	E	Y	Y
sp:O97069:ASSY_DROME	Y	T	S	N	D	R	S	T	D	S	E	E	Y	Y
sp:P22768:ASSY_YEAST	Y	T	S	N	D	R	S	T	D	S	E	E	Y	Y

	whole protein		domain-based		contact-based	
protein	ident	simil	ident	simil	ident	simil
PDB:2nz2:chainA:P00966	1.0	1.0	1.0	1.0	1.0	1.0
tr:Q5T6L4:Q5T6L4_HUMAN	1.0	1.0	1.0	1.0	1.0	1.0
sp:P00966:ASSY_HUMAN	1.0	1.0	1.0	1.0	1.0	1.0
sp:P09034:ASSY_RAT	0.98	0.99	0.98	0.99	1.0	1.0
sp:P16460:ASSY_MOUSE	0.97	0.99	0.97	0.99	1.0	1.0
tr:Q3UJ34:Q3UJ34_MOUSE	0.97	0.99	0.97	0.99	1.0	1.0
tr:J3QNG0:J3QNG0_MOUSE	0.97	0.99	0.97	0.99	1.0	1.0
sp:O97069:ASSY_DROME	0.53	0.83	0.55	0.84	0.93	0.95
sp:P22768:ASSY_YEAST	0.49	0.81	0.51	0.83	0.93	0.95

CG1315 (UniProt:O97069) annotation

Pathway: Amino-acid biosynthesis; L-arginine biosynthesis; L- arginine from L-ornithine and carbamoyl phosphate: step 2/3.

Pathway: Nitrogen metabolism; urea cycle; (N(omega)-L- arginino)succinate from L-aspartate and L-citrulline: step 1/1.

Subunit: Homotetramer.

(Information from UniProt)
